# Corynoline Alleviates Osteoarthritis Development via the Nrf2/NF-*κ*B Pathway

**DOI:** 10.1155/2022/2188145

**Published:** 2022-07-28

**Authors:** Sunlong Li, Yifeng Shi, Shuhao Zhang, Haobin Li, Ziyang Ye, Jiangqian Kong, Weijun Hong, Yiting Tu, Jiaping Ren, Zaher Meftah, Chenglong Xie, Xiangyang Wang, Xiaolei Zhang

**Affiliations:** ^1^Department of Orthopaedics, The Second Affiliated Hospital and Yuying Children's Hospital of Wenzhou Medical University, Wenzhou, Zhejiang Province, China; ^2^Key Laboratory of Orthopaedics of Zhejiang Province, Wenzhou, Zhejiang Province, China; ^3^The Second School of Medicine, Wenzhou Medical University, Wenzhou, Zhejiang Province, China; ^4^The First School of Medicine, Wenzhou Medical University, Wenzhou, Zhejiang Province, China

## Abstract

**Purpose:**

OA is a multifactorial joint disease in which inflammation plays a substantial role in the destruction of joints. Corynoline (COR), a component of *Corydalis bungeana Turcz.*, has anti-inflammatory effects.

**Materials and Methods:**

We evaluated the significance and potential mechanisms of COR in OA development. The viabilities of chondrocytic cells upon COR exposure were assessed by CCK-8 assays. Western blot, qPCR, and ELISA were used to assess extracellular matrix (ECM) degeneration and inflammation. The NF-*κ*B pathway was evaluated by western blot and immunofluorescence (IF). Prediction of the interacting proteins of COR was done by molecular docking, while Nrf2 knockdown by siRNAs was performed to ascertain its significance. Micro-CT, H&E, Safranin O-Fast Green (S-O), toluidine blue staining, and immunohistochemical examination were conducted to assess the therapeutic effects of COR on OA in destabilization of medial meniscus (DMM) models.

**Results:**

COR inhibited ECM degeneration and proinflammatory factor levels and modulated the NF-*κ*B pathway in IL-1*β*-treated chondrocytes. Mechanistically, COR bound Nrf2 to downregulate the NF-*κ*B pathway. Moreover, COR ameliorated the OA process in DMM models.

**Conclusion:**

We suggest that COR ameliorates OA progress through the Nrf2/NF-*κ*B axis, indicating COR may have a therapeutic potential for OA.

## 1. Introduction

Generally, osteoarthritis (OA) is marked with unbearable pain, restricted mobility, and disability, which is known as an irrecoverable, chronic, and degenerative disease [1, 2]. OA has become pervasive around the world due to the aging population, accidental injury, and elevated obesity occurrences. To date, approximately 27 million Americans and more than 250 million people worldwide suffer from this illness [3, 4]. Progresses in biological tissue engineering have improved treatment outcomes and recovery of OA [5]. Stem cell treatment is being evaluated as a potential option for OA treatment [6, 7]. Moreover, joint replacement surgery is exorbitant [8]. Therefore, development of new drugs that can effectively alleviate the symptoms of OA is of utmost importance. We evaluated the significance and efficacy of COR in OA management.

It is well known that inflammatory factors can control the development of OA [9]. Interleukin-1*β* (IL-1*β*) levels are high in OA and are positively related to disease advancement [10, 11]. Inducible nitric oxide synthase 2 (iNOS), interleukin-6 (IL-6), cytochrome C oxidase subunit 2 (COX-2), and tumor necrosis factor-*α* (TNF-*α*), whose expressions are increased by high IL-1*β* levels, seriously damage metabolic transformation of the ECM in knee joints. The ECM of cartilage modulates the biomechanical characteristics of the cartilage, which is mainly composed of aggrecan and type II collagen [12, 13]. However, suppression of IL-1*β* or its receptor demonstrated a treatment potential for OA [14, 15].

In addition, nuclear factor kappa-light-chain-enhancer or the NF-*κ*B pathway regulates inflammatory reaction as well as IL-1*β*-stimulated catabolism in chondrocytic cells [16]. Stimulated by IL-1*β*, a phosphate group is labeled for proteasome degradation and coupled to an NF-*κ*B inhibitor *α* (I*κ*B*α*). This causes the release of p65 protein from its inhibitory complex, which is then translocated into the nucleus [16, 17] and leads to activation of targeted proinflammatory responses or catabolizes downstream genes [18, 19].

Nrf2 is substantially involved in cell homoeostasis, which is known as a significant transcription factor. In reality, Nrf2 activates anti-inflammatory, antiapoptotic, and antioxidant signaling pathway to keep homeostasis [20]. There is a vital interaction between Nrf2 and NF-*κ*B, which is required to hold cell homeostasis as well as promote the inflammatory environment [21, 22]. Amusingly, Nrf2 activation depressed IL-1*β*-associated NF-*κ*B activations in the human chondrocytes [23]. In addition, if the level of Nrf2 and NF-*κ*B is out of balance, it could lead to numerous diseases, such as neurodegeneration [24]. Therefore, the Nrf2/NF-*κ*B axis is considered essential as a target for OA treatment. Heme oxygenase 1 (HO-1) is a pivotal effector for Nrf2-dependent cellular response [25]. In addition, Nrf2/HO-1 is involved in OA progression [26].

Corynoline (COR), a component of *Corydalis bungeana Turcz.*, has anti-inflammatory effects and inhibits the NF-*κ*B pathway [27, 28]. Moreover, COR protects cells by activating Nrf2 signaling [29]. In consideration of what is reported about COR, its anti-inflammatory effects may be a potential mechanism for protection against OA, particularly via the Nrf2/NF-*κ*B pathway.

We evaluated the protective effect of COR on OA and assessed its significance on Nrf2/NF-*κ*B signaling. Our findings will inform the development of treatment strategies for OA.

## 2. Materials and Methods

### 2.1. Ethical Statement

Guiding principles used for surgery, treatment, and postoperative care closely followed the recommendations of the Animal Care and Use Committee at the Wenzhou Medical University.

### 2.2. Reagents and Antibodies

COR (purity ≥ 98%) and Cell Counting Kit-8 (CCK-8) were bought from MedChemExpress (New Jersey, USA) while the recombinant human IL-1*β* was procured from Novoprotein (Shanghai, China). Dimethylsulfoxide (DMSO) as well as collagenase type II was procured from Sigma-Aldrich (St Louis, MO, USA). Primary antibodies are ADAMTS-5, aggrecan, and collagen II (Abcam, Cambridge, MA, USA); GAPDH, iNOS, MMP-3, and MMP-13 (Proteintech, Wuhan, China); and I*κ*B*α*, p-p65, p-I*κ*B*α*, COX-2, p65, Nrf2, and HO-1 (Cell Signaling Technology, Beverly, MA, USA). Secondary antibodies such as goat anti-mouse and anti-rabbit IgG-HRP came from Bioworld (OH, USA), and Alexa Fluor®594-labelled goat and Alexa Fluor®488-labelled anti-rabbit IgG (H+L) were obtained from Jackson ImmunoResearch (West Grove, PA, USA). The cell culture media were obtained from Gibco (Grand Island, USA), while 4′,6-diamidino-2-phenylindole (DAPI) was obtained from Yeasen Biotechnology (Shanghai, China).

### 2.3. Primary Mouse Chondrocyte Extraction

To extract primary chondrocytes from 20 immature C57BL/6 mice, cartilages of knee were cut into 1 mm^3^ cubes before PBS washed 3 times and then digested for 4 h in collagenase II (2 mg/ml) at 37°C. Cell cultures were done in DMEM/F12 containing 10% FBS as well as 1% of antibiotics followed by incubation in a humid environment at 5% CO_2_ and 37°C. Replacement of the media was done once every two days. Second passage chondrocytes were used in the following experiments.

### 2.4. Experimental Design

For in vitro experiment, seeded chondrocytes (in a six-well plate) were grown for 24 hours to achieve a 50-70% confluence. Then, they were treated using IL-1*β* (10 ng/ml), either alone or with varying concentrations of COR (0, 2, and 4 *μ*M) for 24 hours. Next, western blot, PCR, or IF was conducted. Transfection of cells with siRNA (50 nM) was done using the Lipofectamine 2000 siRNA transfection reagent (Thermo Fisher) for 24 hours. Nrf2-siRNA and vehicle-siRNA came from RiboBio (Guangzhou, China).

In vivo, after surgical DMM, COR groups (15 mg kg^−1^ and 30 mg kg^−1^) were intragastrically administered with COR once per day for 8 weeks. Control group mice received the same amount of physiological saline. After 8 weeks (postoperation), mice were sacrificed and cartilage tissue samples obtained for histological assessments.

### 2.5. Assessment of Cell Viabilities

Cellular survival was evaluated by the Cell Counting Kit-8 (CCK-8; MedChemExpress, USA). Chondrocytes (50,000 cells/cm^2^) were cultured in 96-well culture plates and incubated for 1 day after which they were treated using IL-1*β* and COR for 24 h. Treated cells were washed with PBS, which were exposed to DMEM/F12 (100 *μ*l) as well as CCK-8 (10 *μ*l), in 37°C for one hour. Finally, absorbance at 450 nm was read by a microplate reader (Thermo, USA).

### 2.6. NO, PGE2, TNF-*α*, and IL-6 Measurement

Chondrocytes were incubated with 10 ng/ml IL-1*β* supplemented with COR for 24 h. NO, TNF-*α*, PGE2, and IL-6 expression levels in supernatants were then evaluated. Controls were incubated with IL-1*β* only. Concentration of NO was evaluated using Griess reagent (Beyotime, Shanghai, China). PGE2, IL-6, and TNF-*α* levels were determined using ELISA Kits obtained from R&D Systems (Minneapolis, MN).

### 2.7. Intracellular ROS Level Determination

ROS levels in chondrocytes were assessed using a Reactive Oxygen Species Assay Kit (Beyotime, China). After treatment, cells were washed using PBS and incubated with serum-free medium containing 10 *μ*M DCFH-DA for 20 min at 37°C. Cells were then washed with PBS and observed under a fluorescence microscope (Nikon, Japan). Quantification of fluorescence intensities was done by ImageJ software (Bethesda, USA).

### 2.8. Real-Time PCR

TRIzol (Invitrogen, USA) was used for total RNA extraction, followed by cDNA synthesis using 1000 ng RNA and a commercial kit (MBI Fermentas, Germany) of PrimeScript-RT reagent (Japan). SYBR Premix Ex Taq (Takara, Japan) and the CFX96 Real-Time PCR system (Bio-Rad Laboratories, USA) were used for amplification. Cycle thresholds (Ct) of detected transcripts were normalized by GAPDH, the control. Lastly, relative gene levels were detected by the 2^−△△^Ct method [30]. Finally, primers used in this experiment were described in the previous research [19].

### 2.9. Western Blotting

Chondrocytic cell lysis was performed using the radio immunoprecipitation assay (RIPA) (Meilunbio, China) buffer and phenylmethanesulfonyl fluoride (PMSF, 1 mM) (NCM Biotech, China) and then centrifuged for 15 min at 12,000 rpm and 4°C. BCA protein assessment kit (Meilunbio, China) was used for protein level quantification. Then, the protein (40 *μ*g) was separated on a sodium dodecyl sulfate polyacrylamide gel (8–12% (*w*/*v*), Beijing Solarbio Science & Technology Co., Ltd., China) electrophoresed and transferred to polyvinylidene fluoride membranes (Merck Millipore, USA). Blocking of the membranes was done within 2 h using 5% nonfat milk and incubation for 12 h in the presence of primary antibodies against ADAMTS-5 (1 : 1000), aggrecan (1 : 800), COX-2 (1 : 1000), p65 (1 : 1000), MMP-3 (1 : 1000), iNOS (1 : 1000), I*κ*B-*α* (1 : 1000), MMP-13 (1 : 1000), p-I*κ*B-*α* (1 : 800), p-p65 (1 : 1000), HO-1 (1 : 1000), Nrf2 (1 : 1000), or GAPDH (1 : 2000) done at 4°C. Next, after TBST washed 3x, incubation was done with secondary antibodies for two hours at room temperature (RT). Then, the Plus reagent (Invitrogen, NY, USA) and the ChemiDoc XRS+ Gel Imaging System (Bio-Rad, CA, USA) were used to visualize the membranes, after another wash 3 times with TBST. Finally, quantification of protein band intensities was done by ImageJ 2.1 (Bethesda, USA), and standardized using GAPDH.

### 2.10. Immunofluorescence

Chondrocytes were planted in six-well plates and treated as mentioned previously, fixed in paraformaldehyde (4% (*v*/*v*)) for 15 min after being rinsed using PBS, permeabilized for 10 min using Triton X-100 (0.1%) dissolved in PBS, blocked using 10% bovine serum albumin for one hour at 37°C, treated with primary antibodies for COL II (1 : 100), p65 (1 : 100), or Nrf2 (1 : 100) at 4°C overnight, incubated with Alexa Fluor®594-conjugated or Alexa Fluor®488-labelled secondary antibodies (1 : 200) for one hour at RT, and 4′,6-diamidino-2-phenylindole (DAPI) stained for 5 minutes. Then, fluorescence microscopy (Olympus Inc., Tokyo, Japan) was performed to analyze 5 random fields selected from per slide. Finally, the fluorescence intensity was quantified by observers who had been blinded to sample groups with ImageJ 2.1 (Bethesda, USA). ImageJ 2.1 was used to quantify the expression of protein by integrated optical density (IOD).

### 2.11. Molecular Modelling

The crystal structure of Keap1-Nrf2 target protein (PDB ID: 3WN7) comes from the protein database (https://www.rcsb.org/structure). The compound structure (corynoline) comes from the PubChem database (https://pubchem.ncbi.nlm.nih.gov/).

The treated compound is utilized as a small molecule ligand, and the protein target is used as a receptor. The center position, length, width, and height of the Grid Box are determined according to the interaction between the small molecule and the target. Finally, batch docking was performed through AutoDock, and molecular docking findings analyzed. Binding effects of the compound and protein were visualized using PyMOL 2.1 software. In the calculation process, the Lamarckian genetic algorithm is used for molecular docking. The algorithm is as follows: 150 population, maximum 25 million energy evaluations, maximum number of 2000s, 0.8 crossover rate, a mutation rate of 0.02, 50 independent docking runs, and according to combine free energy to evaluate the final docking structure.

After molecular docking, first visualize the top-ranked compounds (PyMOL) and analyze the interaction mode of the compound with the target protein and the interaction with the active site residues (such as hydrogen bond interaction, *π*-*π* interaction, and hydrophobic interaction) [31].

### 2.12. Animal Model

Eight-week-old C57BL/6 male mice from the Animal Center of Chinese Academy of Sciences (Shanghai, China) were randomized into DMM, sham, DMM+COR (15 mg/kg), and DMM+COR (30 mg/kg) groups. Osteoarthritis mouse models were established by surgical DMM. Anaesthesia of mice in the DMM and DMM+COR groups was done by intraperitoneal administration of pentobarbital (2% (*w*/*v*)). Next, right knee joint capsules were incised medial to the patellar tendon, after which medial meniscotibial ligaments were transected using microsurgical scissors. Arthrotomy without medial meniscotibial ligament transection was conducted in sham group mice. Sham group and DMM group mice were pretreated by intragastric administration of CMC-Na solution (0.5%) each day, while DMM+COR group mice were pretreated COR (15 mg/kg or 30 mg/kg in 0.5% CMC-Na solution) [29, 32].

### 2.13. Micro-CT Analysis

The knee joints of mice were fixed in 4% paraformaldehyde for 24 h and then scanned in a 16 mm scanning tube with 10.5 mm^3^ volume at 55,000 V, 180 mA, with a 115 min acquisition time. To avoid dehydration, joints were wrapped in napkins dampened with PBS while scanning. The data were analyzed using Skyscan software.

### 2.14. Histopathology

For 24 h, sample fixation was done in 4% (*v*/*v*) paraformaldehyde, decalcified using 10% ETDA and sliced into 5 *μ*m thick sections. Staining of sample slides was done using H&E, Safranin O-Fast Green (S-O), and toluidine blue. Cellular structure and morphologies of cartilage as well as subchondral bone were microscopically (Olympus Inc.) evaluated by blinded histologists and scored as per the recommended osteoarthritis scoring system [33].

### 2.15. Immunohistochemical Analysis

Sections were deparaffinized, incubated in the presence of H_2_O_2_ (3%) for 10 minutes, and washed thrice using PBS. They were incubated for 20 minutes in the presence of 0.1% trypsin and washed thrice using PBS. Next, blocking of the sections was done using goat serum albumin (1% (*w*/*v*)) for one hour at 37°C. Overnight incubation of sections at 4°C was done with primary antibodies against Nrf2 and collagen II, washed thrice using PBS, and incubated in the presence of HRP-conjugated secondary antibodies for two hours at 37°C. Negative control sections were incubated with nonspecific IgG. For observation, at least 3 sections from every specimen were used. Positive cell rate for every section was quantitatively determined by blinded researchers.

### 2.16. Statistical Analysis

Data are shown as means ± standard deviation (SD) from independent experiments. Analyses were done by GraphPad Prism (USA) (one-way ANOVA followed by Tukey's post hoc test). The nonparametric data (OARSI scores) were analyzed using the Kruskal-Wallis *H* test. *p* ≤ 0.05 was statistically significant.

## 3. Results

### 3.1. COR-Associated Cellular Toxicity and Chondrocyte Protection

**Figure 1:**
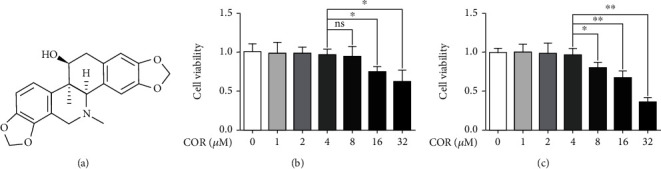
Effects of COR on the viability in IL-1*β*-treated chondrocytes. (a) The chemical structure for COR. (b, c) Cytotoxicity of varying doses of COR on chondrocytes after 24 h and 72 h. Data are averages ± SD of 3 separate experimentations. ^∗^*p* < 0.05, ^∗∗^*p* < 0.01, and ^∗∗∗^*p* < 0.001.

The COR chemical structure is exhibited in Figure 1(a). To determine whether COR is cytotoxic, chondrocytes were given varying COR doses (0, 1, 2, 4, 8, 16, and 32 *μ*M) for 24 or 72 hours before cell survival was measured. According to our result, there was no obvious cytotoxicity when the concentration reached 4 *μ*M. However, COR concentrations > 8 *μ*M significantly reduced cell viability (Figures 1(b) and 1(c)). Therefore, 0, 2, and 4 *μ*M COR concentrations were performed to assess its impact on IL-1*β*-treated chondrocytes in following experiments.

### 3.2. COR Impact on ECM Catabolism and Anabolism in IL-1*β*-Treated Chondrocytes

**Figure 2:**
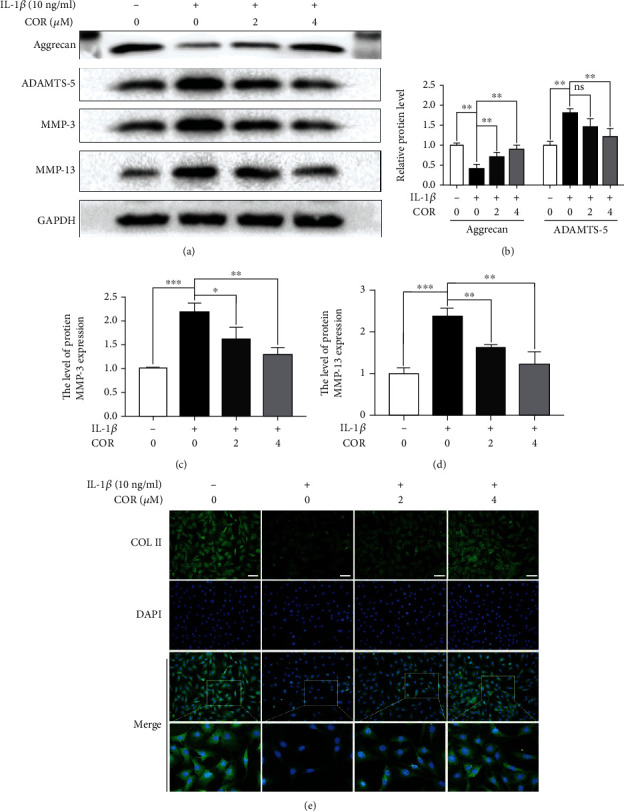
Effect of COR on ECM anabolism as well as catabolism in IL-1*β*-stimulated chondrocytes, with or without COR. (a) ECM catabolic- as well as anabolic-associated protein levels in IL-1*β*- and COR-exposed chondrocytes, compared with control. (b–d) Protein levels were analyzed employing ImageJ. (e) COL II was assessed by fluorescence microscopy, combined with nuclear DAPI staining (scale bar: 50 *μ*m). The values are mean ± SD for *n* = 3. ^∗^*p* < 0.05, ^∗∗^*p* < 0.01, and ^∗∗∗^*p* < 0.001.

Anabolic-related proteins were assessed in COR- as well as IL-1*β*-treated cells to determine the protective effects of COR on anabolism. COL II and aggrecan levels in COR-treated cells were elevated, relative to IL-1*β*-treated cells (Figures 2(a) and 2(e)). Moreover, relative to IL-1*β*, COR strongly suppressed ECM catabolic-related proteins, MMP-13, ADAMTS-5, and MMP-3 (Figures 2(a)–2(d)).

### 3.3. COR Modulates Inflammation Levels in IL-1*β*-Treated Chondrocytes

**Figure 3:**
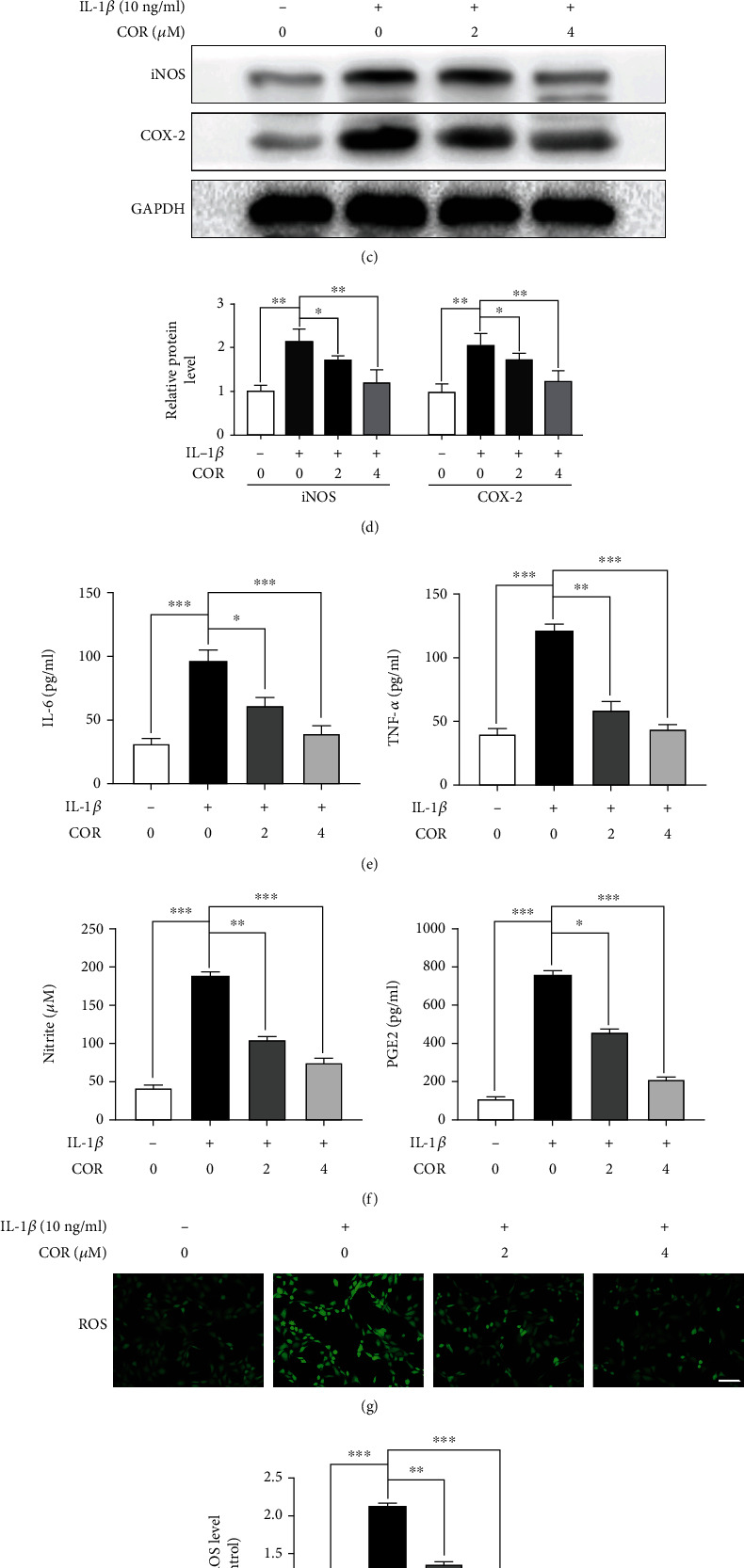
Effects of COR on modulating proinflammatory factor levels in IL-1*β*-stimulated chondrocytes. (a, b) Inflammatory-associated transcripts were analyzed by PCR. (c) Inflammatory-associated protein levels were determined by western blot. (d) Protein quantities were analyzed employing ImageJ. (e, f) The levels of IL-6, TNF-*α*, PGE2, and NO in the cell culture supernatants were measured by ELISA. (g, h) ROS activities were detected using the DCFH-DA probe (scale bar: 50 *μ*m). The values are averages ± SD for 3 separate experimentations. ^∗^*p* < 0.05, ^∗∗^*p* < 0.01, and ^∗∗∗^*p* < 0.001.

Based on PCR and ELISA, TNF-*α* as well as IL-6 levels were significantly elevated upon IL-1*β* administration, but were suppressed by COR (Figures 3(a) and 3(e)). Similarly, employing PCR as well as western blot analyses, we demonstrated significant IL-1*β*-associated elevations of COX-2 as well as iNOS, which were inhibited by COR (Figures 3(b)–3(d)). These findings imply that COR inhibits proinflammatory pathways. ELISA and Griess result demonstrated that COR inhibited IL-1*β*-induced secretion of endogenous nitric oxide (NO) and prostaglandin E2 (PGE2) (Figure 3(f)). DCFH-DA fluorescent probe was used to detect ROS levels in the chondrocytes, and the results showed that IL-1*β* treatment induced oxidative stress, while COR attenuated ROS generation (Figures 3(g) and 3(h)).

### 3.4. COR Impact on the NF-*κ*B Pathway in Chondrocytes Treated with IL-1*β*

**Figure 4:**
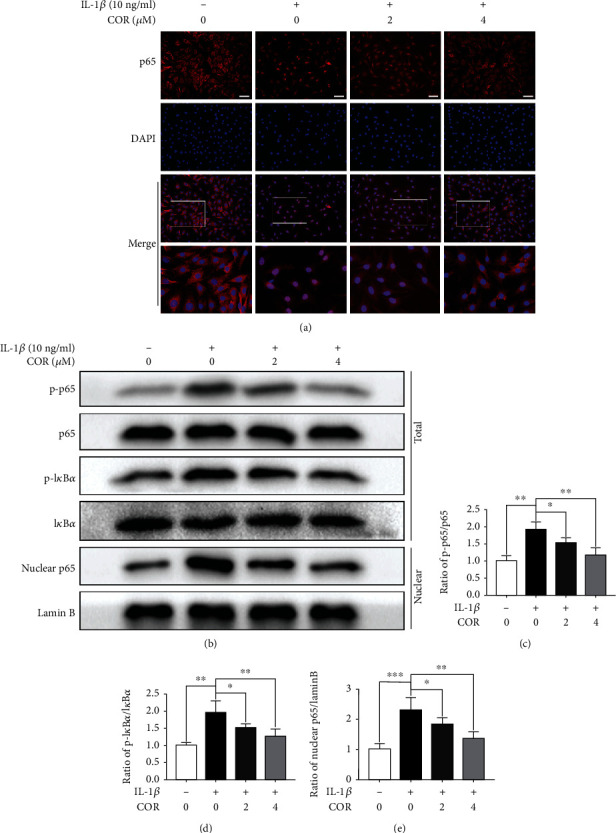
Effect of COR on the NF-*κ*B pathway in IL-1*β*-stimulated chondrocytes, with or without COR treatment. (a) p65 localization as assessed by IF, combined with nuclear DAPI staining (scale bar: 50 *μ*m). (b) Inflammatory-associated protein levels. (c–e) Quantification of inflammation-related proteins was analyzed employing ImageJ. The values are averages ± SD for 3 separate experiments. ^∗^*p* < 0.05, ^∗∗^*p* < 0.01, and ^∗∗∗^*p* < 0.001.

In view of evident inhibition to proinflammatory factors of COR, we further detected whether the protective effect of COR to chondrocytes involved the NF-*κ*B pathway. The IF staining demonstrated that p65 was translocated to the nucleus in IL-1*β*-stimulated chondrocytes. Relatively, COR-treated chondrocytes revealed that p65 was highly localized in cytoplasms (Figure 4(a)). Additionally, compared to cells without treatment, we exhibited a great deal of phosphorylated I*κ*B*α* and p65 in IL-1*β*-treated chondrocytes, suggesting NF-*κ*B pathway activation. Comparatively, the COR-treated cells showed notably low level of phosphorylated p65 as well as I*κ*B*α* (Figures 4(b)–4(d)). The chondrocyte nucleus was further isolated, and the p65 protein level in the nucleus was assessed. Consistent with the IF image, COR repressed existence of p65 in the nucleus (Figures 4(b) and 4(e)). Briefly, these data imply apparent NF-*κ*B pathway inhibition in COR-exposed chondrocytes.

### 3.5. Molecular Docking of COR and Nrf2

The NF-*κ*B pathway is modulated by a variety of upstream regulators, including Nrf2 [26], a potential OA treatment target [34]. We performed molecular docking analysis to establish the possible interactions between COR and Nrf2/NF-*κ*B pathway proteins.

**Figure 5:**
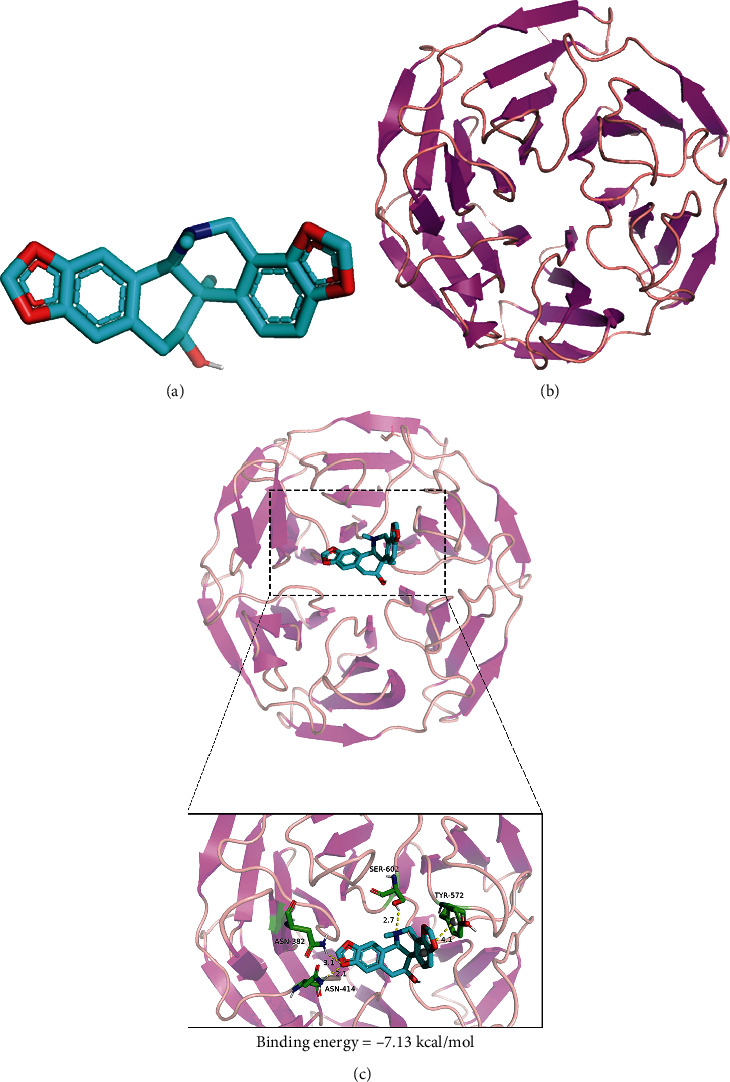
Molecular docking of COR on its Nrf2 binding site. (a) COR model. (b) Nrf2 ribbon model. (c) High interaction energy (-7.13 kcal/mol) between COR and Nrf2. The compound forms strong hydrogen bonds with the active groups of ASN-382, SER-602, and ASN-414. The distances are 3.1 Å, 2.7 Å, and 2.1 Å, respectively.

The Nrf2 protein has a COR binding site (Figures 5(a) and 5(b)). The results of molecular docking show that the binding energy is -7.13 kcal/mol, and the compound has a strong binding effect with the target protein, because the lower energy of the compound and the target is, the better the binding will be (Figure 5(c)).

The compound formed by the compound and protein after docking is visualized using PyMOL 2.1 software to obtain the binding mode of the compound and the protein. According to the binding mode, the amino acid residues of the compound and the protein pocket can be clearly seen; corynoline and Keap1-Nrf2 target protein binding active amino acid residues are ASN-382, SER-602, ASN-414, and TYR-572. The compound forms strong active groups with ASN-382, SER-602, and ASN-414. The hydrogen bond distances are 3.1 Å, 2.7 Å, and 2.1 Å, respectively, which are smaller than the traditional hydrogen bond of 3.5 Å. The combination is strong and plays an important role in stabilizing small molecule ligands. In addition, the benzene ring of corynoline and the TYR-572 amino acid of the active pocket of the protein form a strong *π*-*π* conjugated interaction (Figure 5(c)).

In summary, these interactions can improve the stability of the compound in the Keap1-Nrf2 protein pocket, so the compound is a potentially active small molecule.

### 3.6. COR Modulates the NF-*κ*B Pathway via Nrf2

**Figure 6:**
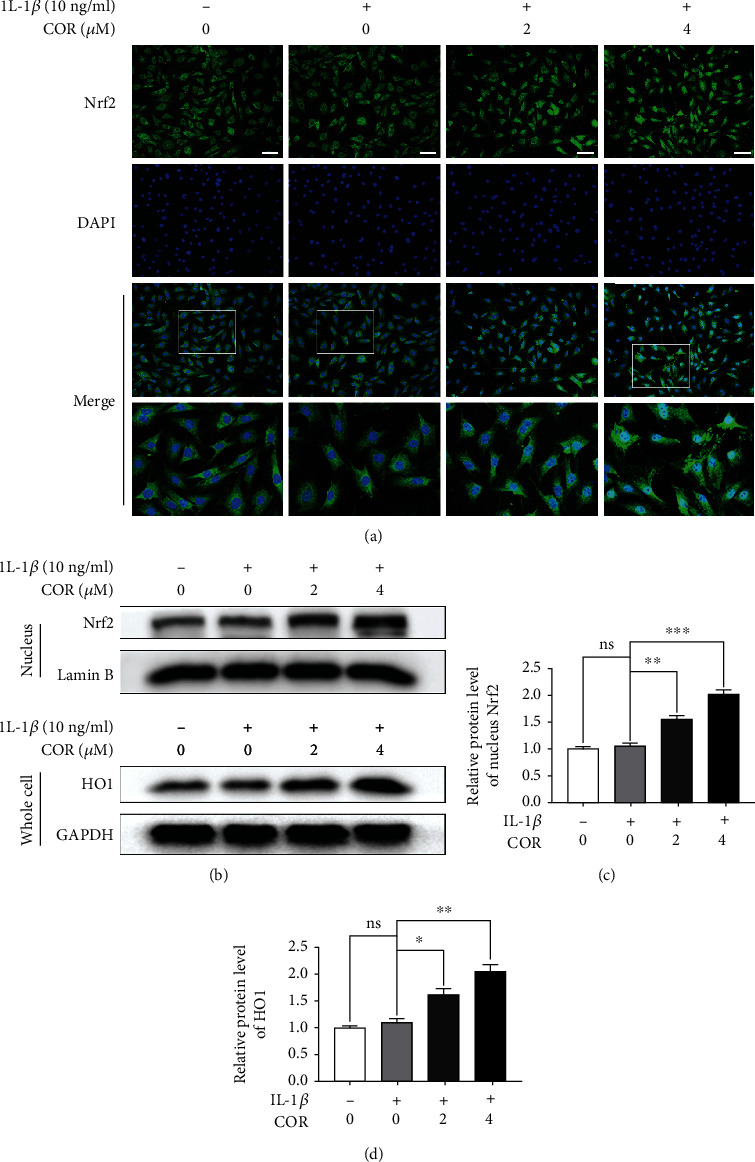
Effect of COR on the Nrf2 pathway in IL-1*β*-stimulated chondrocytes. (a) Nrf2 was assessed by IF combined with nuclear DAPI staining (scale bar: 50 *μ*m). (b–d) Nrf2 levels in nuclear fractions as well as downstream HO-1 in the entire extract were assessed by western blot. Protein quantification was analyzed employing ImageJ. The values are mean ± SD for *n* = 3. ^∗^*p* < 0.05, ^∗∗^*p* < 0.01, and ^∗∗∗^*p* < 0.0001.

**Figure 7:**
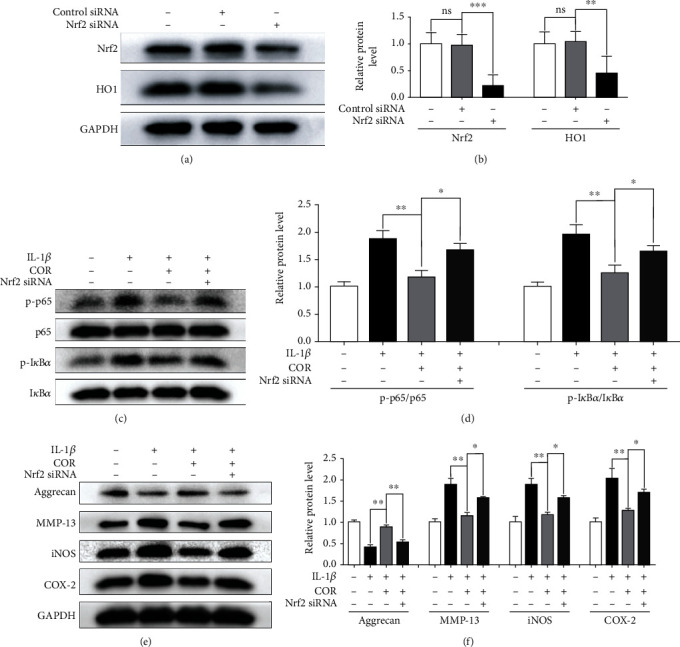
The lack of Nrf2 reduces COR impact on IL-1*β*-stimulated chondrocytes. Nrf2 knockdown was done using siRNA in IL-1*β*-stimulated chondrocytes. (a, b) Detection of Nrf2 as well as HO-1 protein levels. Protein was quantificated by ImageJ. (c, d) Phosphorylated as well as unphosphorylated p65 and I*κ*B*α* forms were detected by western blot. Protein quantification was analyzed using ImageJ. (e, f) Assessment of the Nrf2 pathway-associated protein levels. The values are mean ± SD for *n* = 3. ^∗^*p* < 0.05, ^∗∗^*p* < 0.01, and ^∗∗∗^*p* < 0.001.

We evaluated Nrf2 localization by IF to verify that the Nrf2 pathway is involved in COR-associated cytoprotection of IL-1*β*-stimulated chondrocytic cells. Nrf2 significantly translocated to the nucleus under COR administration (Figure 6(a)). Similarly, the level of Nrf2 in the nucleus of COR-administrated chondrocytes was significantly elevated, compared with IL-1*β* alone. In addition, Nrf2-targeted HO-1 protein levels in whole cell extracts were increased, compared to IL-1*β*-stimulated chondrocytes (Figures 6(b)–6(d)). Next, we used siRNA to knockdown Nrf2 in the separated chondrocytic cells. Using western blot, we demonstrated that Nrf2 and HO-1 proteins were underexpressed when Nrf2 was absent, compared with the negative control (Figures 7(a) and 7(b)). Moreover, Nrf2 deficiency resulted in the production of more phosphorylated I*κ*B*α* and p65 proteins than the control (Figures 7(c) and 7(d)). We showed that COR regulated the NF-*κ*B pathway via its modulation of Nrf2. In addition, our data exposed that COR treatment elevated the level of aggrecan protein, compared with IL-1*β* alone (Figure 7(e)). Conversely, Nrf2 deficiency markedly inhibited the amount of aggrecan protein, while MMP13 protein was significantly raised (Figure 7(f)). These findings imply that COR protects the ECM by regulating Nrf2. Moreover, COR administration lowered COX-2 and iNOS protein levels, while lack of Nrf2 raised the expression levels of both proteins (Figures 7(e) and 7(f)), indicating COR modulates inflammation through Nrf2.

### 3.7. COR Alleviates OA Progression in DMM Mouse Models

**Figure 8:**
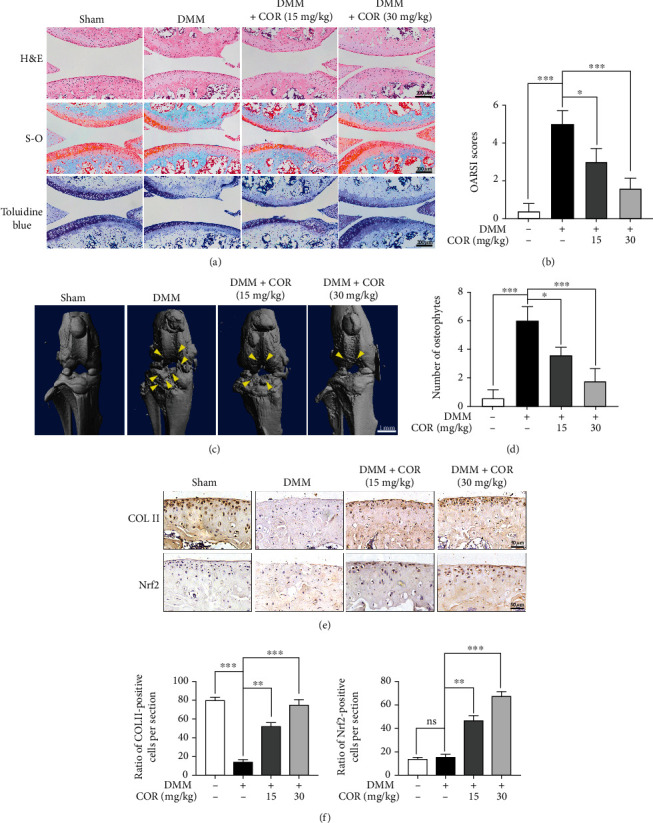
COR alleviated OA progression in mouse models. (a) Typical H&E, Safranin O-Fast Green (S-O), and toluidine blue staining of cartilage from different experimental groups (sham, DMM, DMM+COR15mg/kg, and DMM+30 mg/kg groups) (scale bar: 200 *μ*m). (b) OARSI scores for cartilage in the 4 groups. (c) 3D reconstruction images of micro-CT scanning of the knees and osteophytes (yellow arrow) (scale bar: 1 mm). (d) The number of osteophytes. (e) The expressions of COL II as well as Nrf2 in the mouse cartilage were assessed using immunohistochemical staining (scale bar: 50 *μ*m). (f) The quantification of COL II and Nrf2. The values are mean ± SD. ^∗^*p* < 0.05, ^∗∗^*p* < 0.01, and ^∗∗∗^*p* < 0.001. *n* = 5 for micro-CT images and quantification of staining; *n* = 3 for IHC staining quantification.

The DMM-induced mouse OA models were used to determine if COR exerts protective effects on OA development in vivo. The histopathological examination demonstrated that no pathological changes were found in the heart, kidney, and liver of the mice in each experimental group, compared to the control group (Figure S1). H&E, S-O, and toluidine blue staining indicated that COR treatment reduced the degree of superficial cartilage destruction, loss of proteoglycan, and cartilage erosion in the OA models in a dose-dependent manner (Figure 8(a)). Consistently, OARSI scores for the DMM group were substantially elevated relative to the sham group, while scores for the DMM+COR groups (15 and 30 mg/kg) were lower than those of the DMM group (Figure 8(b)). To investigate structural changes of bones in the COR-treated OA models, three-dimensional imaging was carried out using micro-CT. 3D reconstruction revealed more osteophytes in the DMM group whereas COR could suppress osteophyte formation in OA progression (Figures 8(c) and 8(d)). Moreover, immunohistochemical staining showed that COL II levels were suppressed in the DMM group, while the COR could reverse the COL II level in the DMM+COR group (Figures 8(e) and 8(f)). In addition, COR was shown to suppress Nrf2 levels (Figures 8(e) and 8(f)). These findings indicate that COR suppresses cartilage degradation in OA mice in a dose-dependent manner.

## 4. Discussion

OA is related to severe discomfort, restricted mobility, and disability, which is a widespread, incurable, and degenerating disease that affects joints. Effective treatment options for OA have yet to be developed. OA is characterized by severe joint inflammation. As a therapeutic drug, COR demonstrated hope in inhibiting inflammation in various tissues. Thus, we investigated if COR can regulate OA progress. Our results showed that COR inhibited the NF-*κ*B pathway by mediating Nrf2 to slow down the progress of OA. This potential discovery can provide a further reference for the treatment of OA development in the future.

COR has been shown to exhibit protective effects against inflammation-related diseases, such as upper respiratory tract infections [35], acute lung injury [29], inflammation of the cardiovascular system [36], and allergic rhinitis [37]. We exposed isolated chondrocytic cells to a series of effective and harmless COR concentrations to detect the protective effect of COR on OA. Our study demonstrated that COR inhibited both excess ECM catabolism and inadequate anabolism in IL-1*β*-stimulated chondrocytic cells.

Additionally, inflammation enhances OA development [9], and artificial inhibition of inflammation has been shown to reduce OA progress [38–40]. Zhai et al. documented that COR treatment reduced iNOS as well as COX-2 expression in RAW 264.7 cells significantly [28]. Moreover, another study suggested that COR markedly reduced IL-6 and TNF-*α* levels [37]. We demonstrated that COR presence obviously decreased proinflammatory factor levels in IL-1*β*-stimulated chondrocytic cells, which confirmed previous studies.

The IL-1*β*-mediated inflammatory response was controlled by NF-*κ*B signaling [16]. Our study proved that COR significantly inhibited the NF-*κ*B pathway by suppressing p65 phosphorylation, thereby isolating it in cytoplasms. Consistently, studies have demonstrated apparent COR-mediated inhibition of NF-*κ*B as well as its downstream pathway [41, 42].

In addition, the Nrf2-associated signaling pathway is a main regulator in IL-1-mediated inflammatory response in chondrocytes [22]. Furthermore, the Nrf2 pathway can effectively target the NF-*κ*B pathway thereby delaying OA development [26, 40]. Our study has strongly suggested that COR administration could suppress the advancement of OA by mediating Nrf2 pathway activation. We have demonstrated that COR interacted with residues in the inhibitory interacting pocket of Nrf2. Liu et al. and Yang et al. found that COR inhibited the Nrf2 pathway [35, 36].

The DMM mouse models exhibited abnormal osteophytes, narrow joint-space, and calcification. COR protected against degradation of the cartilage. Meanwhile, our study showed the level of COL II increased in the DMM+COR group, indicating that COR can promote homeostasis of the extracellular matrix. In addition, COR inhibited the level of Nrf2 in OA mice. Consistent with the experimental results in vitro, these phenomena suggested that COR had the potential to treat OA.

**Figure 9:**
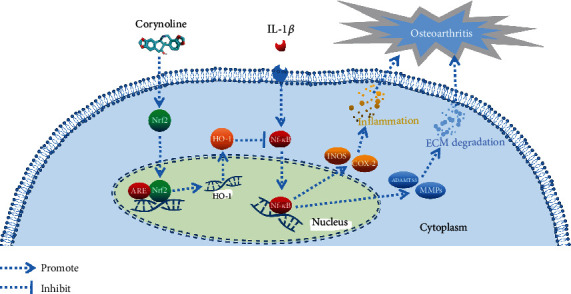
Schematic diagram of the protective effects of COR in OA. COR not only downregulates the iNOS and COX-2 expressions to inhibit inflammation but also decreases ECM degradation by regulating ADAMTS5 and MMPs, via the Nrf2/NF-*κ*B pathway in the OA development.

In conclusion, COR alleviated OA by suppressing inflammation and ECM degradation through the Nrf2/NF-*κ*B pathway (Figure 9).

## Data Availability

The data used to support the findings of this study are included within the article.

## Supplementary Materials

Supplementary 1 Western blots: the raw data of the western blots in the manuscript.

Supplementary 2 Figure S1: biological toxicity results of COR in vivo. (A) Typical H&E staining of the heart, kidney, and liver from different experimental groups (scale bar: 100 or 50 *μ*m).
