# Correction to “Corynoline Alleviates Osteoarthritis Development via the Nrf2/NF‐κB Pathway”

**DOI:** 10.1155/omcl/9783010

**Published:** 2026-03-01

S. Li, Y. Shi, S. Zhang, et al., “Corynoline Alleviates Osteoarthritis Development via the Nrf2/NF‐κB Pathway,” *Oxidative Medicine and Cellular Longevity* 2022 (2022): 2188145, https://doi.org/10.1155/2022/2188145.

In the article titled, “Corynoline Alleviates Osteoarthritis Development via the Nrf2/NF‐κB Pathway,” there was an error in Figure 2. In Figure 2A, the MMP‐3 band is duplicated with ADAMTS‐5. This duplication originated from an error introduced by the publisher during the secondary image editing process. These are two distinct bands. The correct figure is shown below:

Figure 2Effect of COR on ECM anabolism as well as catabolism in IL‐1β‐stimulated chondrocytes, with or without COR. (a) ECM catabolic‐ as well as anabolic‐associated protein levels in IL‐1β‐ and COR‐exposed chondrocytes, compared with control. (b–d) Protein levels were analyzed employing ImageJ. (e) COL II was assessed by fluorescence microscopy, combined with nuclear DAPI staining (scale bar: 50 μm). The values are mean ± SD for *n* = 3.  ^∗^ *p* < 0.05,  ^∗∗^ *p* < 0.01, and  ^∗∗∗^ *p* < 0.001.

**Figure:**
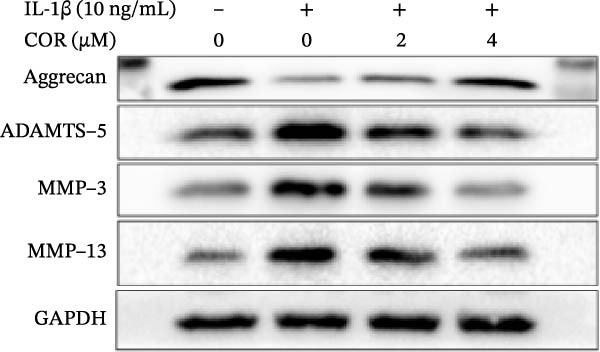
(a)

**Figure:**
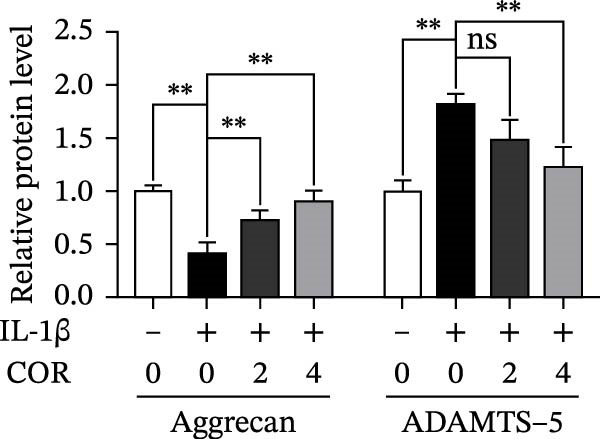
(b)

**Figure:**
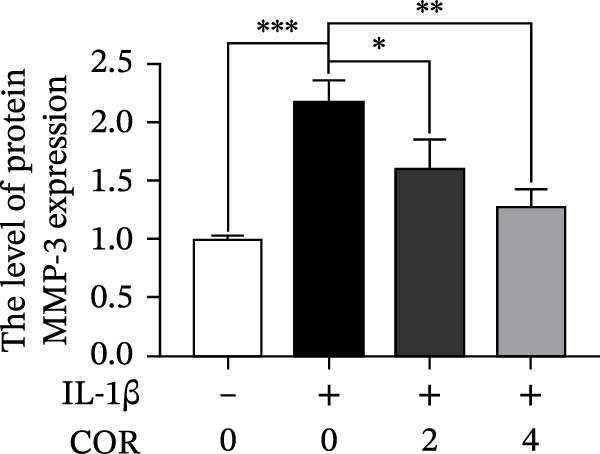
(c)

**Figure:**
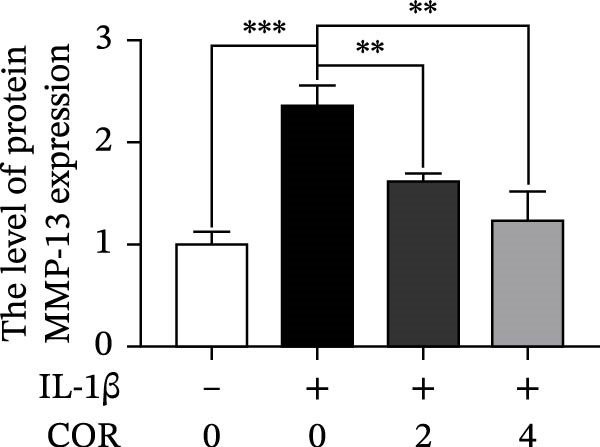
(d)

**Figure:**
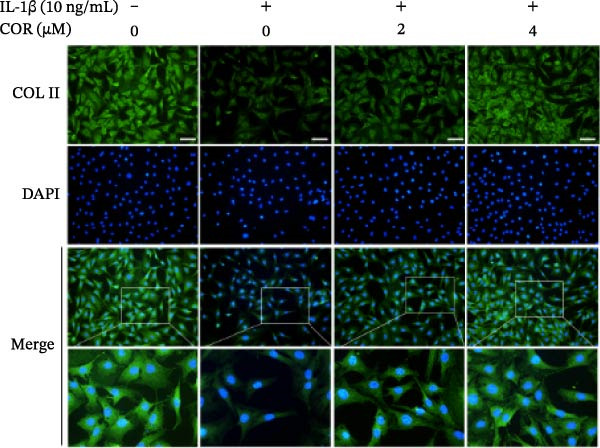
(e)

We apologize for this error.

