# Supplementary material for: Corynoline Alleviates Osteoarthritis Development via the Nrf2/NF-κB Pathway
Source: Oxid Med Cell Longev. 2022 Jul 28;2022:2188145. doi: 10.1155/2022/2188145 (PMC9356246; doi:10.1155/2022/2188145)
Supplement: Supplementary 1 — Western blots: the raw data of the western blots in the manuscript. [file 2188145.f1.docx]

**Figure2**

**
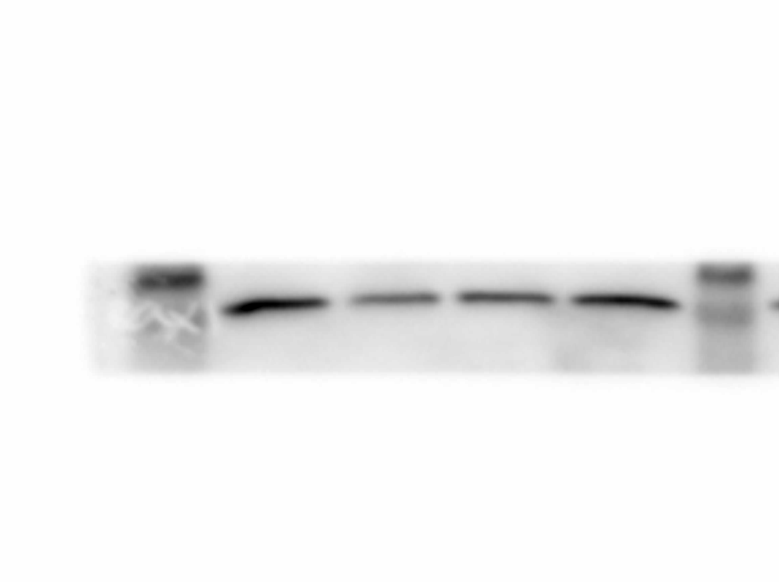
**

**Aggrecan**


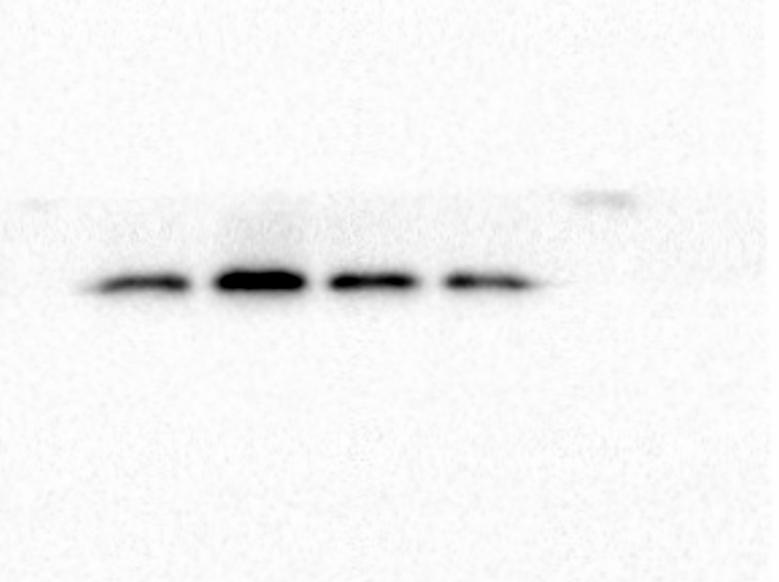


**ADAMTS-5**

**MMP3**
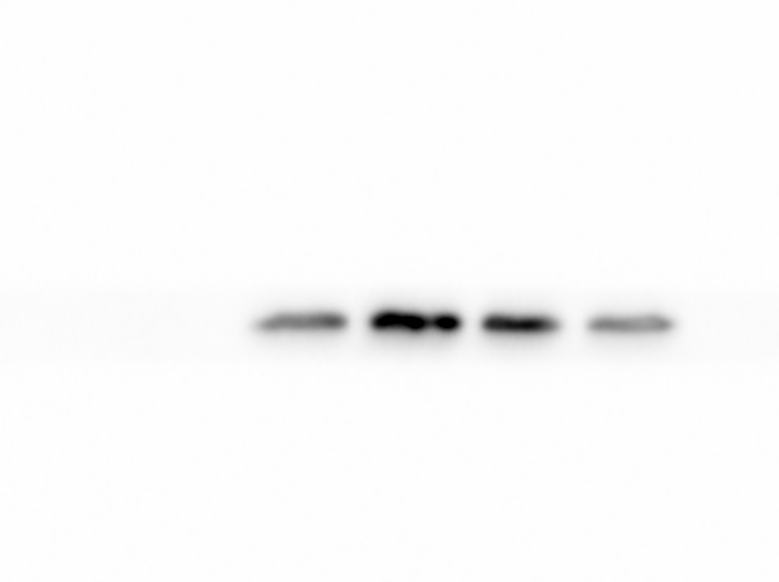


**
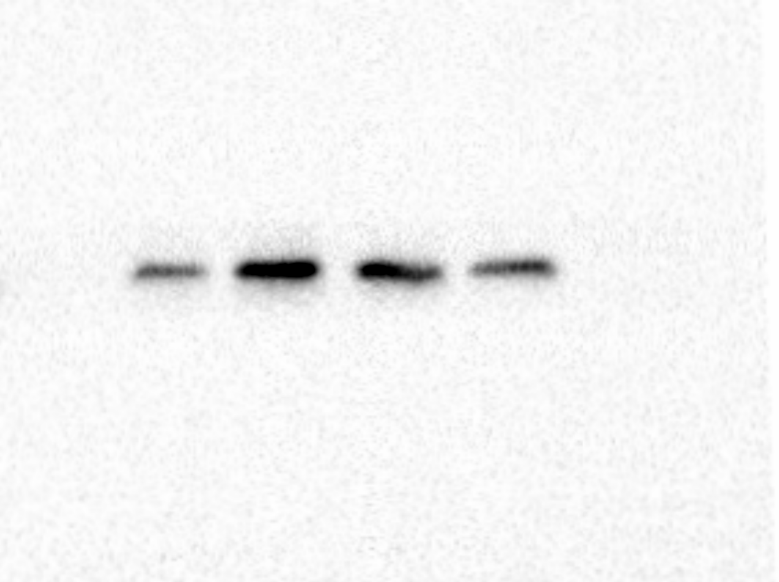
**

**MMP13**

**
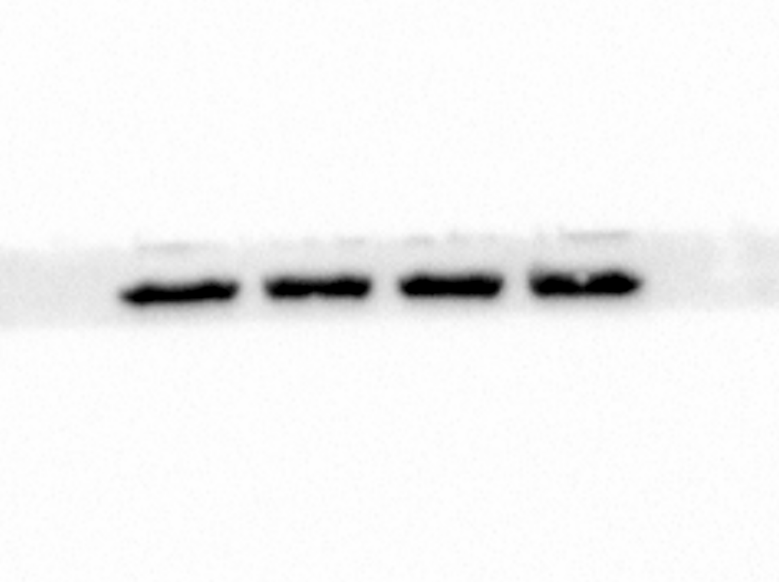
**

**GAPDH**

**Figure3**

**INOS
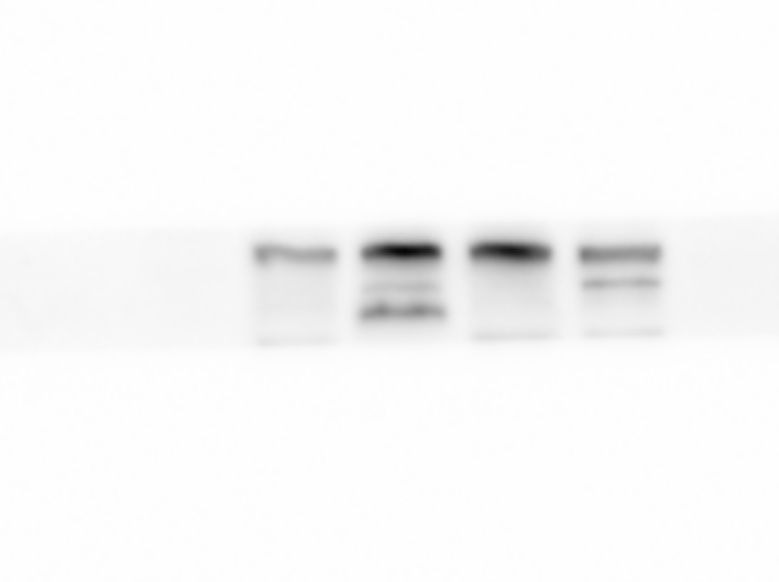
**

**COX2
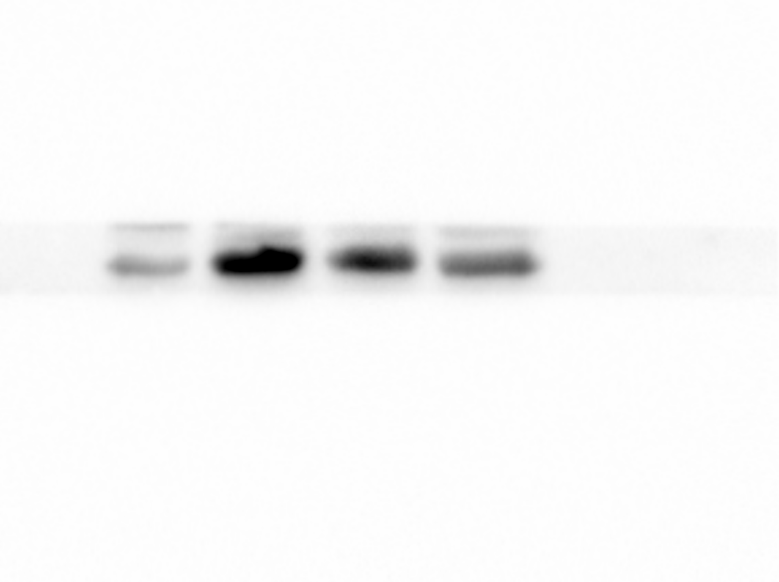
**

**
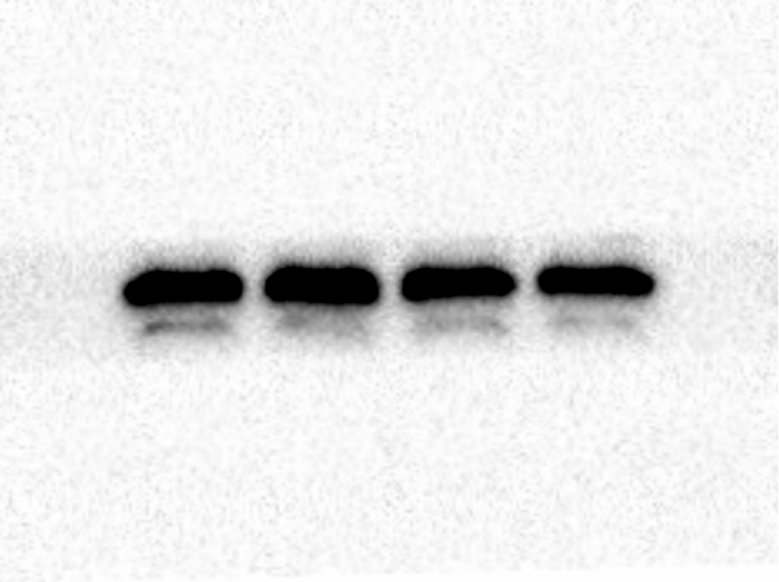
**

**GAPDH**

**Figure4**

**p-p65
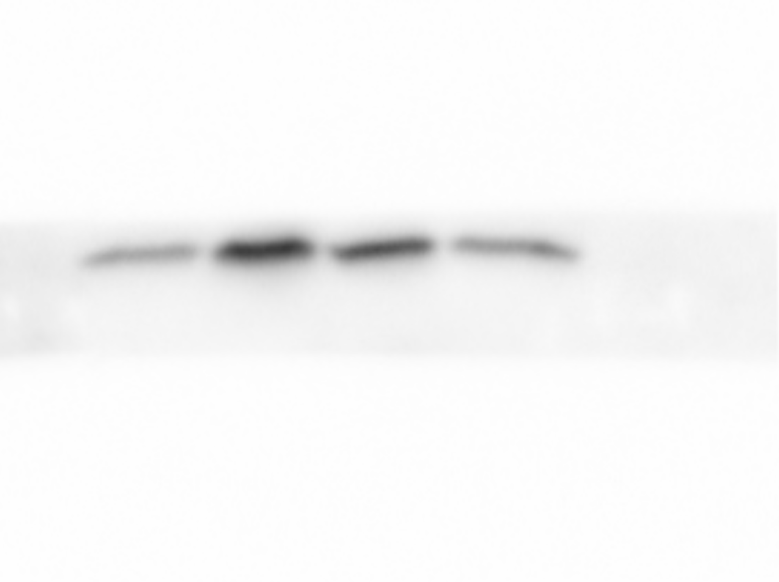
**

**p65
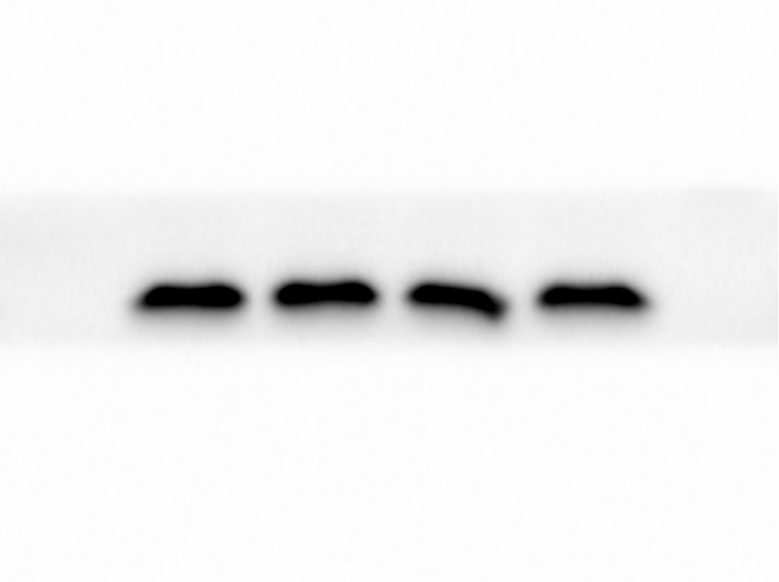
**

**p-IκBα
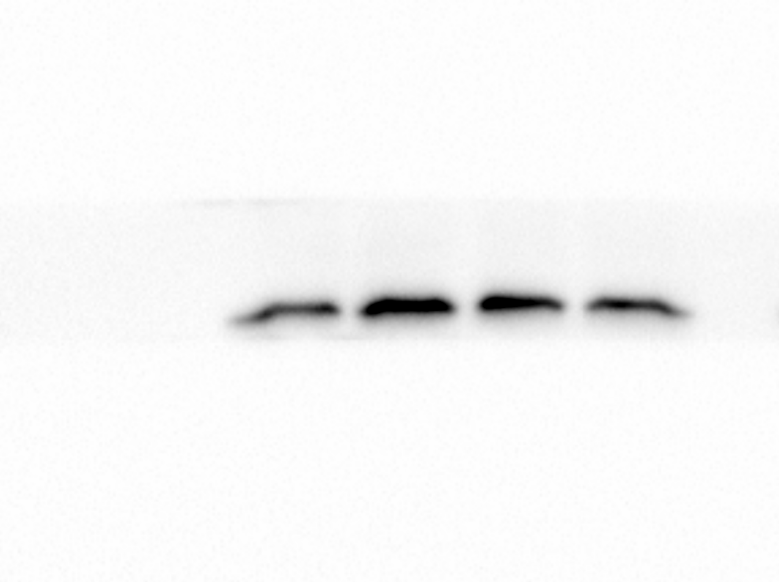
**

**IκBα
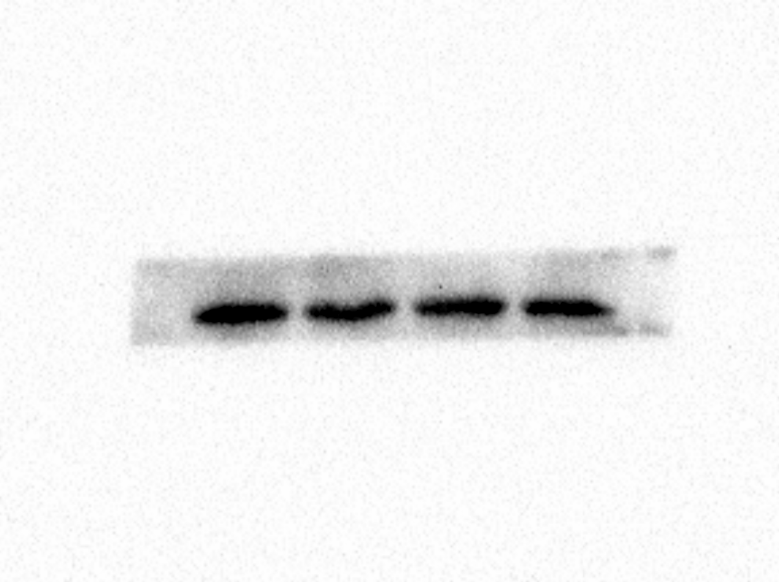
**

**Nuclear p65
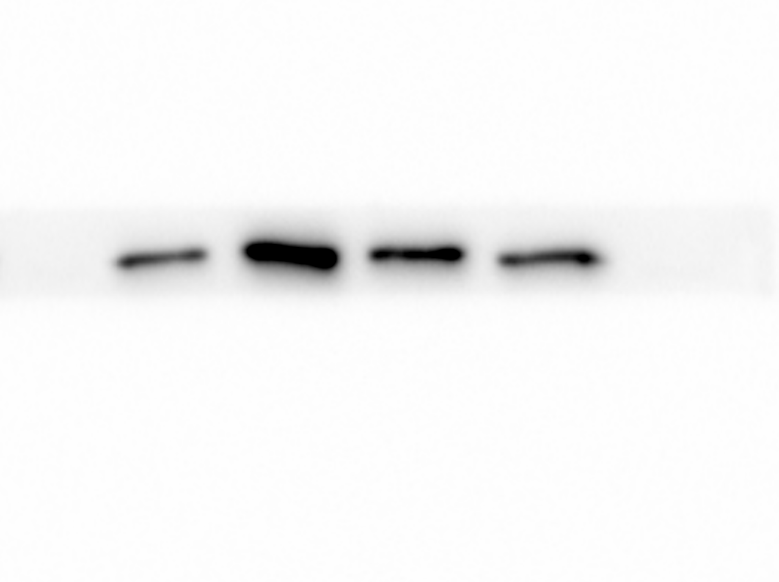
**

**Lamin B
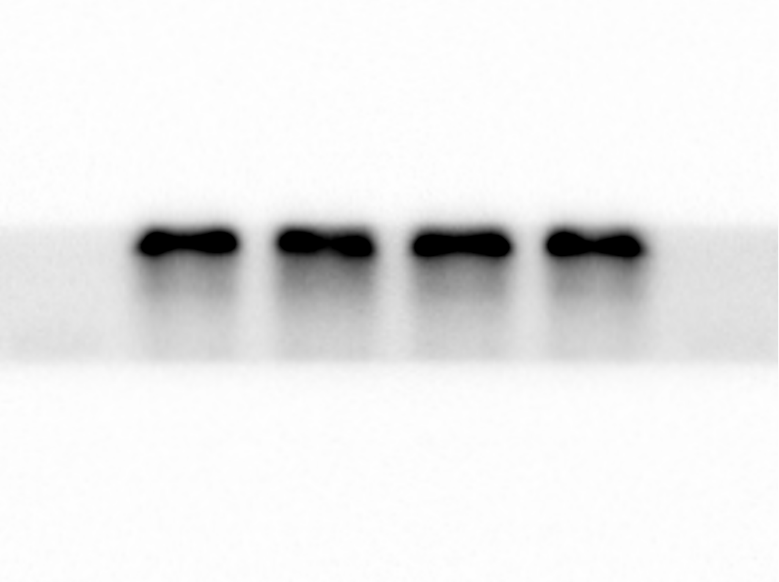
**

**Figure6**

**Nrf2
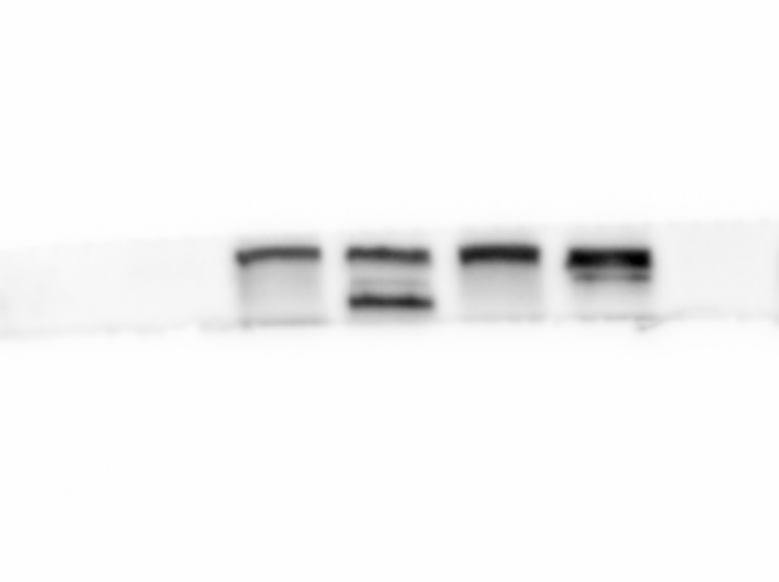
**

**Lamin B
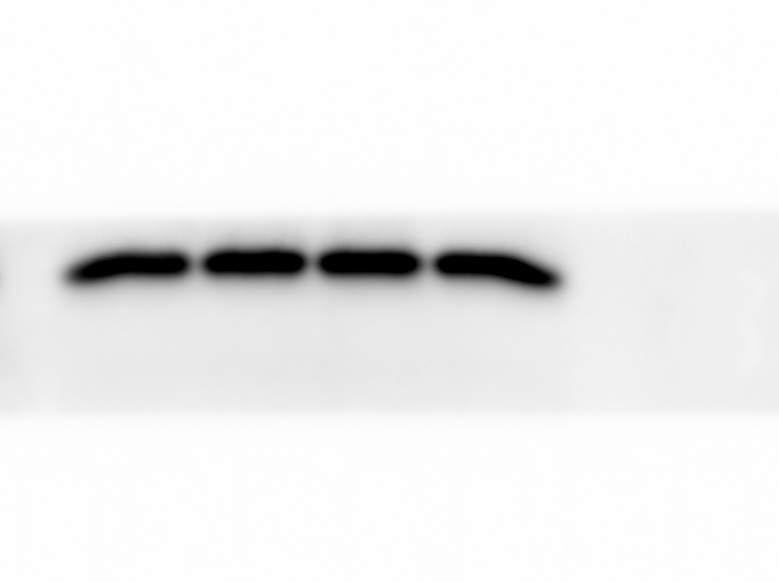
**

**HO1
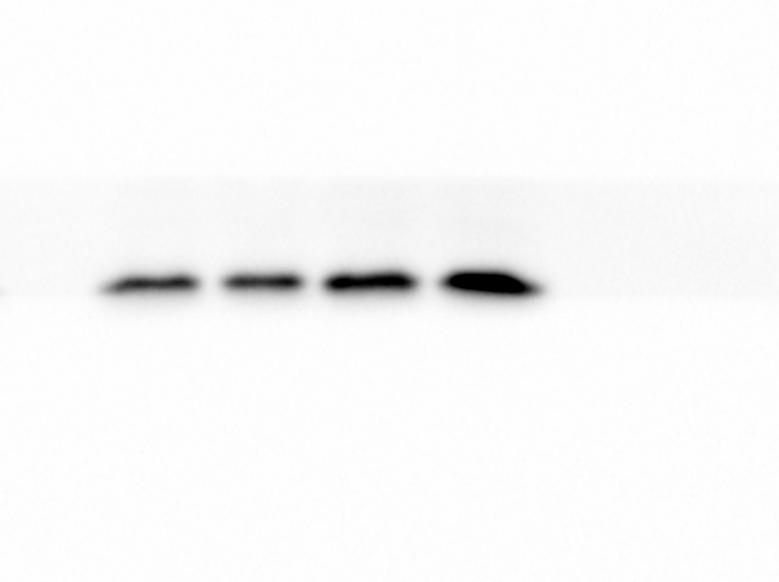
**

**GAPDH
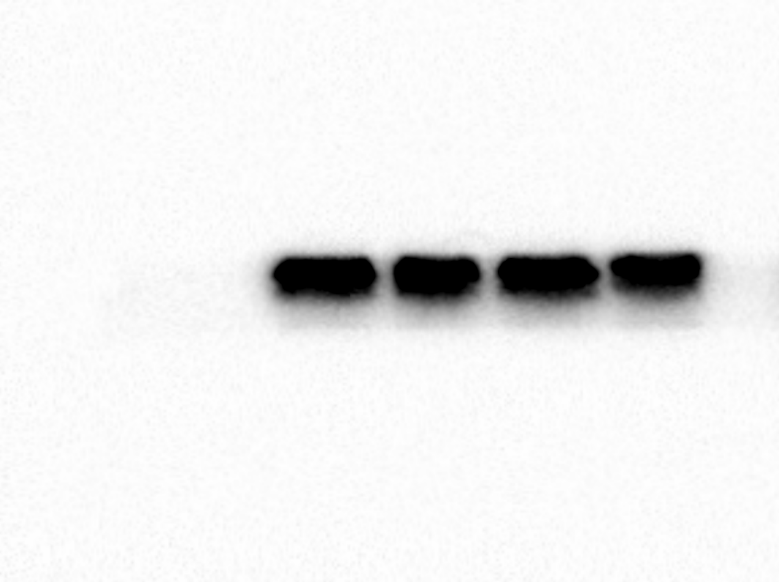
**

**Figure7**

**Nrf2
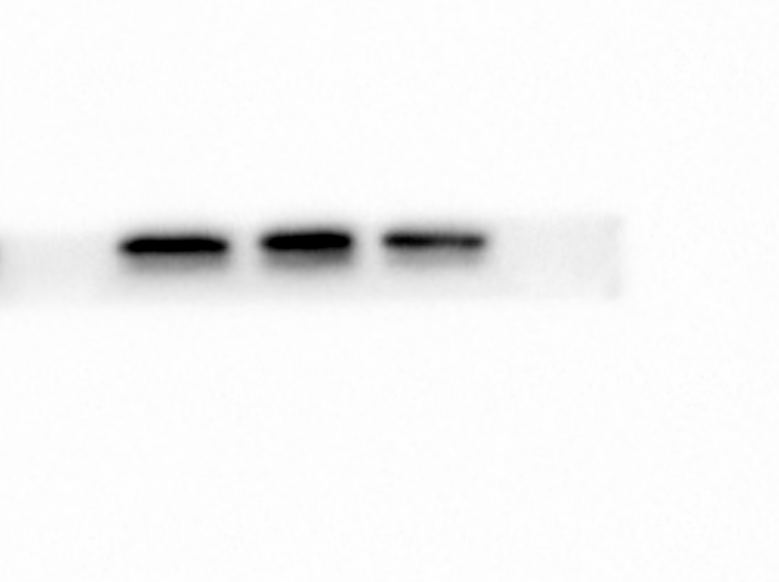
**

**HO1
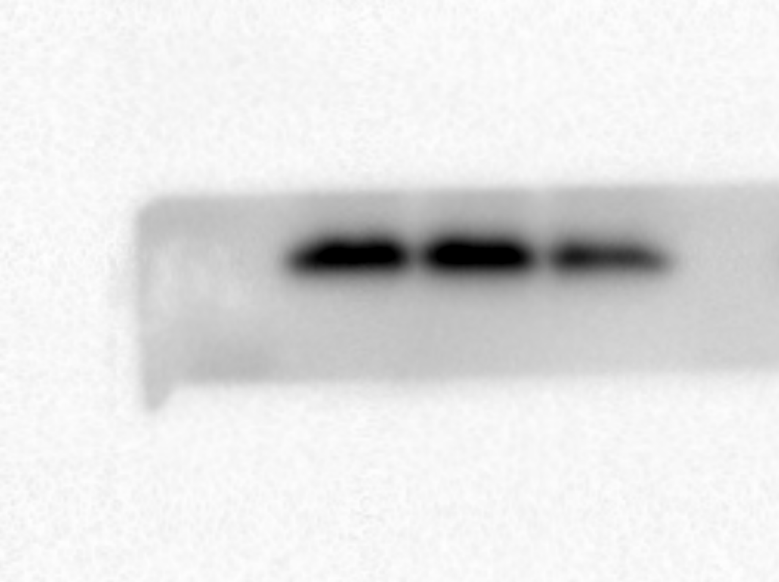
**

**GAPDH
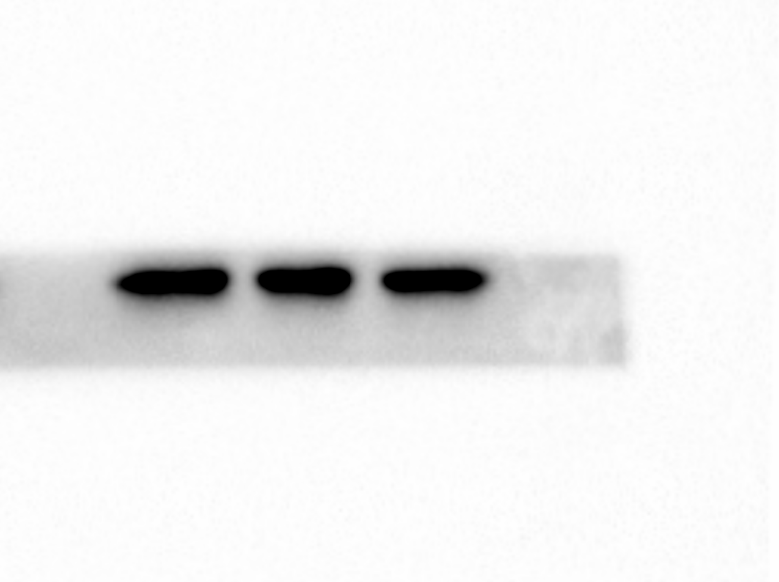
**

**p-p65
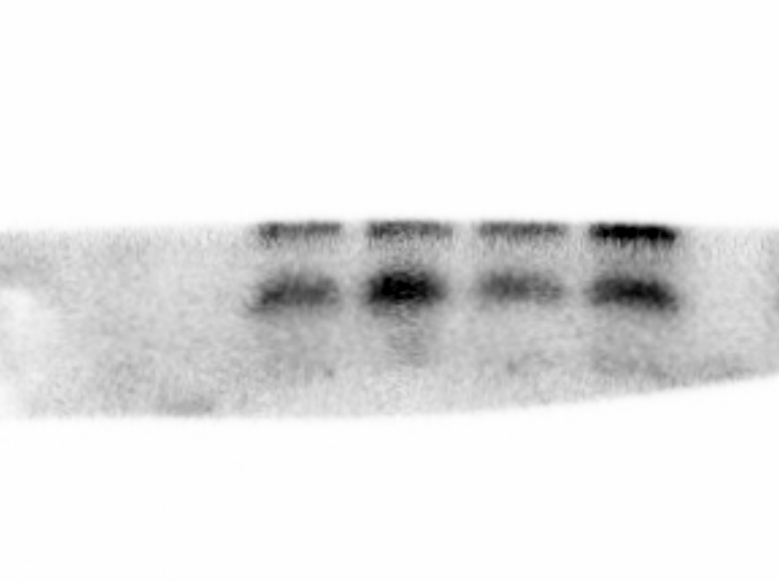
**

**p65
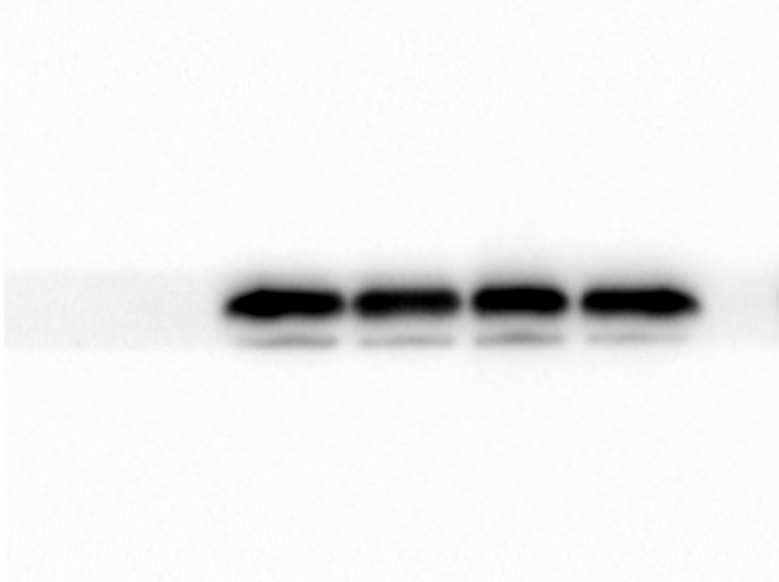
**

**p-IκBα
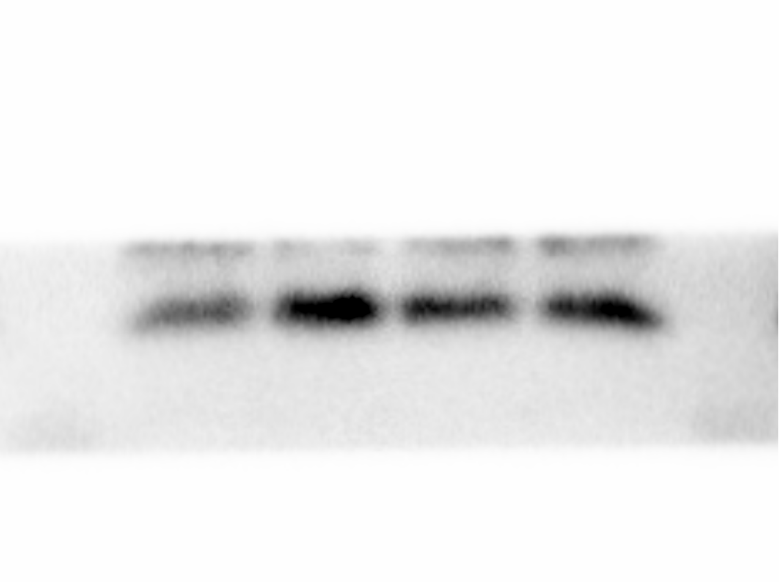
**

**IκBα
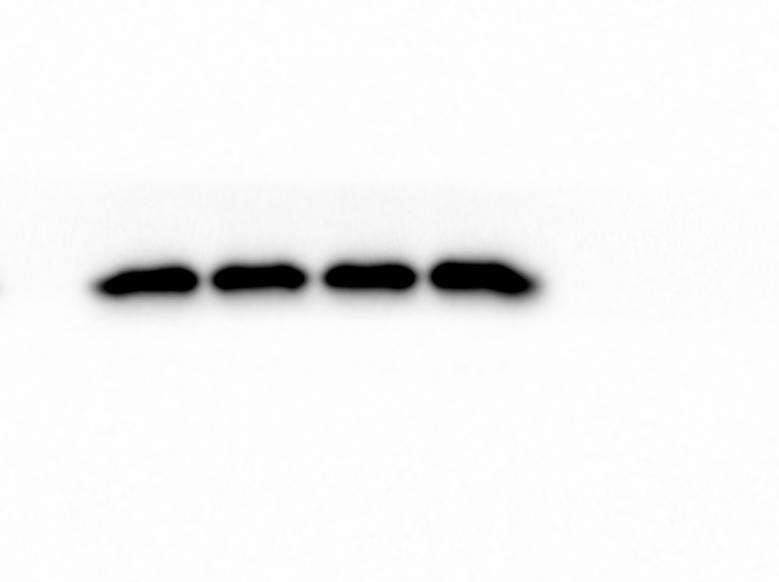
**

**Aggrecan
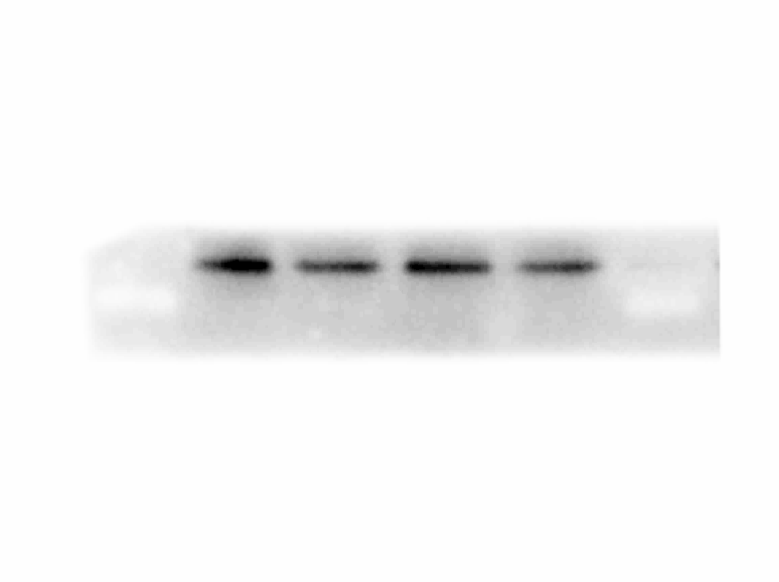
**

**MMP13
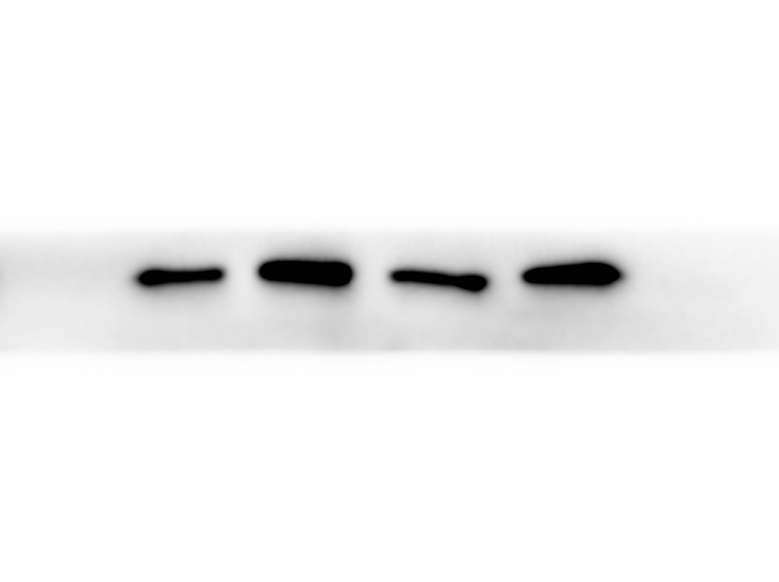
**

**
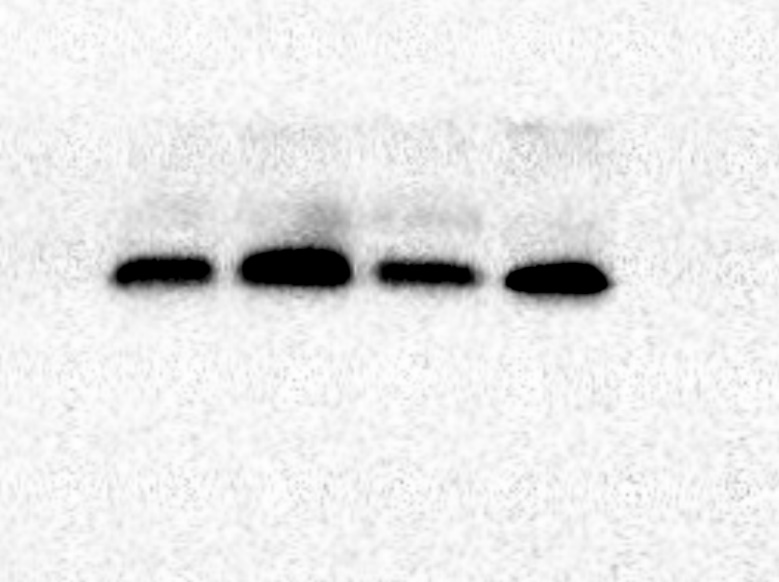
**

**INOS**

**COX2
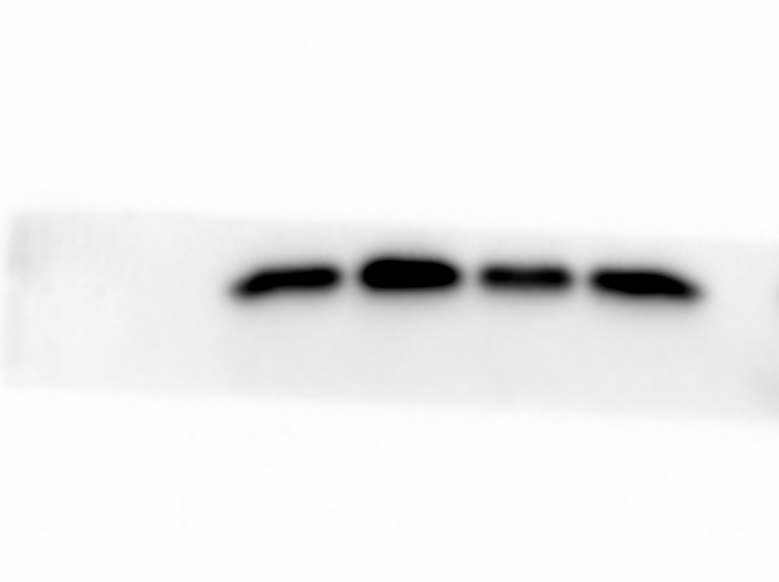
**

**GAPDH
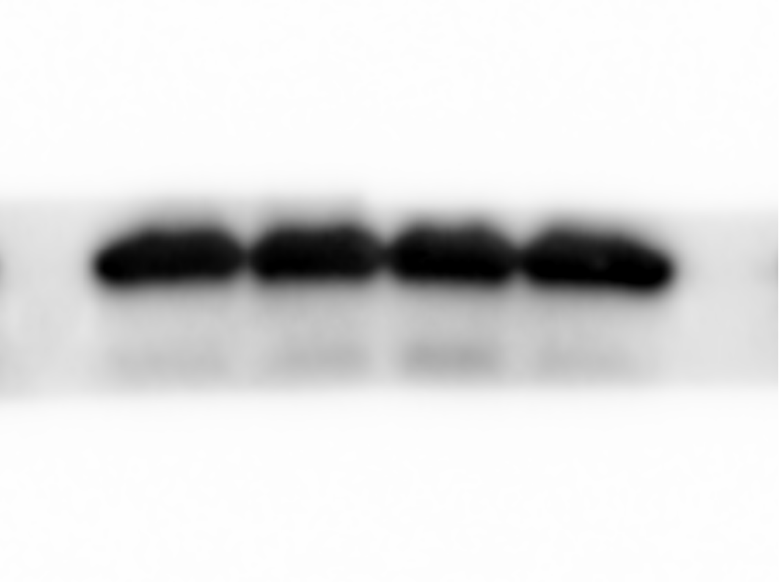
**
