# Supplementary material for: Corynoline Alleviates Osteoarthritis Development via the Nrf2/NF-κB Pathway
Source: Oxid Med Cell Longev. 2022 Jul 28;2022:2188145. doi: 10.1155/2022/2188145 (PMC9356246; doi:10.1155/2022/2188145)
Supplement: Supplementary 2 — Figure S1: biological toxicity results of COR in vivo. (A) Typical H&E staining of the heart, kidney, and liver from different experimental groups (scale bar: 100 or 50 μm). [file 2188145.f2.docx]

**Supplementary Figure**

**
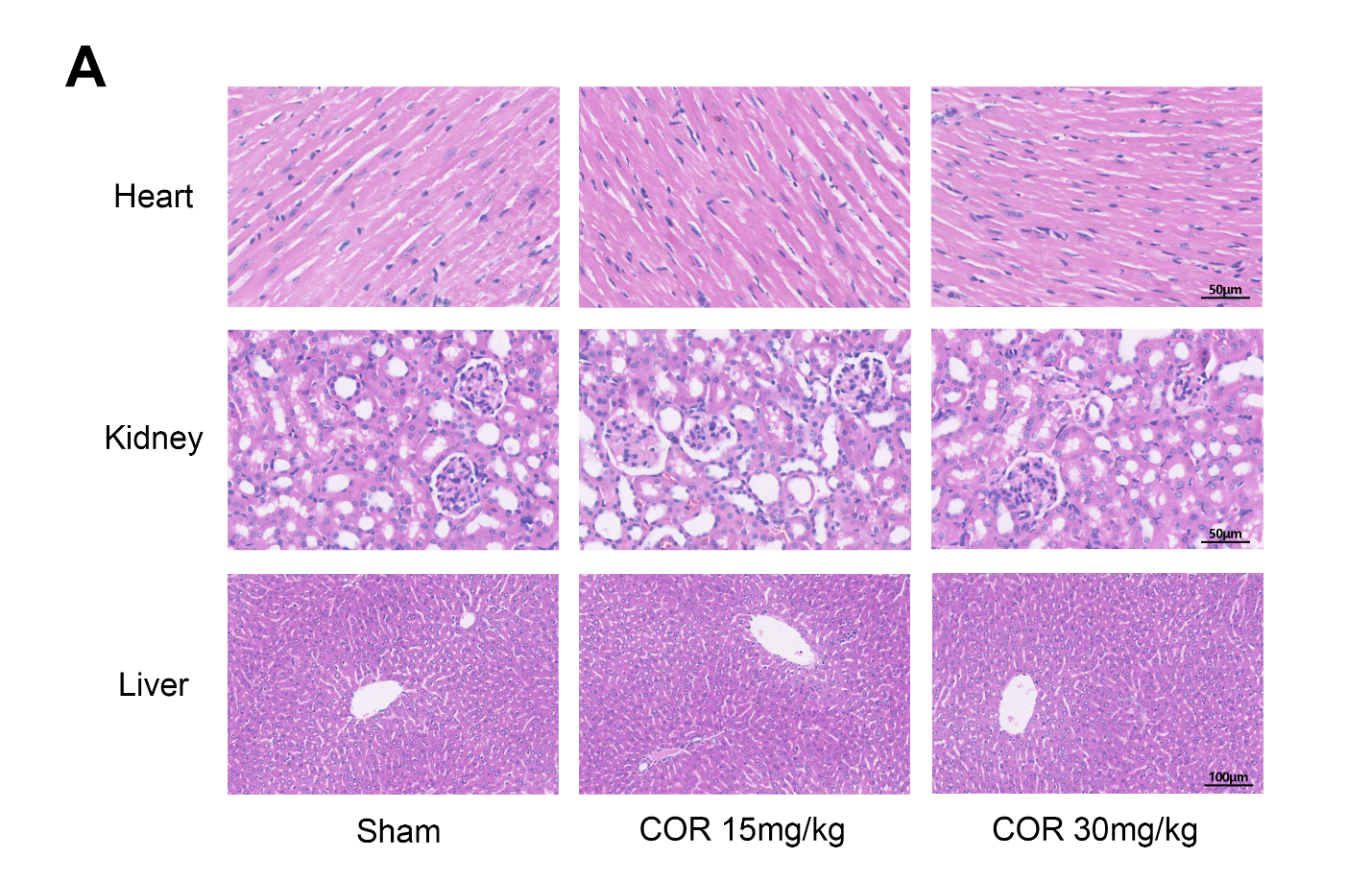
**

**Figure S1.** Biological toxicity results of COR in vivo. (A) Typical H＆E staining of heart, kidney and liver from different experimental groups (scale bar: 100 or 50μm).
